# Patterns of home care and community support preferences among older adults with disabilities in China: a latent class analysis

**DOI:** 10.1186/s12877-023-03830-4

**Published:** 2023-03-03

**Authors:** Feng Xiao, Songmei Cao, Mingzhao Xiao, Liling Xie, Qinghua Zhao

**Affiliations:** 1 Department of Nursing, the First Affiliated Hospital of Chongqing Medical University, Chongqing, China; 2 Department of Urology, the First Affiliated Hospital of Chongqing Medical University, Chongqing, China; 3 Department of Nursing, First Branch of the First Affiliated Hospital of Chongqing Medical University, Chongqing, China

**Keywords:** Home care, Older adults, Community support, Latent class analysis, Preference

## Abstract

**Background:**

Ageing in place is the preferred choice for most older adults worldwide. The role of the family as a core care resource has diminished as a result of changes in family structure, thus extending the responsibility for caring for older adults from within the family to outside it and requiring considerably more support from society. However, there is a shortage of formal and qualified caregivers in many countries, and China has limited social care resources. Therefore, it is important to identify home care patterns and family preferences to provide effective social support and reduce government costs.

**Methods:**

Data were obtained from the Chinese Longitudinal Healthy Longevity Study 2018. Latent class analysis models were estimated using Mplus 8.3. Multinomial logistic regression analysis was adopted to explore the influencing factors with the R3STEP method. Lanza’s method and the chi-square goodness-of-fit test were used to explore community support preferences among different categories of families of older adults with disabilities.

**Results:**

Three latent classes were identified based on older adults with disabilities’ characteristics (degree of disability, demand satisfaction), caregivers’ characteristics (length of providing care, care performance) and living status: Class 1- mild disability and strong care (46.85%); Class 2- severe disability and strong care (43.92%); and Class 3- severe disability and incompetent care (9.24%). Physical performance, geographic region and economic conditions jointly influenced home care patterns (*P* < 0.05). Home visits from health professionals and health care education were the top two forms of community support that were most preferred by the older adults with disabilities’ families (residual > 0). Families in the Class 3 subgroup preferred personal care support more than those in the other two subgroups (*P* < 0.05).

**Conclusion:**

Home care is heterogeneous across families. Older adults’ degrees of disability and care needs may be varied and complex. We classified different families into homogeneous subgroups to reveal differences in home care patterns. The findings can be used by decision-makers in their attempts to design long-term care arrangements for home care and to adjust the distribution of resources for the needs of older adults with disabilities.

## Background

Longevity is an important human achievement, with people today living twice as long as those born in 1900 [1]. However, the “expansion of morbidity” theory hypothesizes that reduced mortality results in more frail older adults surviving with health problems, thus worsening their overall health which is known as the “failure of success (living longer)” [2]. These factors generate great challenges for the health guarantee system, social care services and family support from society. They place pressure not only on China but also on many other countries worldwide, especially developing countries [3].

In 1991, the United Nations Principles for Older Persons stated that older adults should live in their own homes for as long as possible [4]. Despite economic and cultural differences, ageing in place is the universal preference for most older people worldwide [5–8]. However, along with the process of industrialization, the rapid socioeconomic transition led to a series of changes in household size, demographics, and residential structures. The role of the family as a core care resource has diminished, resulting in the responsibility of caring for frail older adults being extended from within the household to outside of it [9]. China implements the “90–7-3 model,” which means that 90 percent of all older adults receive the care they need in their homes, 7 percent receive care at community-based hospitals and health centres, and 3 percent receive care at nursing homes [7]. However, like other countries, China is experiencing a shortage of formal home care providers. In this study, we defined “home care” as informal care that is provided in home-based settings, mainly by family members and other informal (i.e., unpaid) caregivers, and supplemented by formal (i.e., paid) services and support as they are available and affordable.

There are approximately 40.63 million older adults with disabilities, accounting for 18.3% of the older adult population in China [10]. The oldest old sub-population is growing much faster than any other age group, and they are the most likely to need help. Meanwhile the largest majority of the oldest old aged 80 years and above living in private households, and very small proportion live in the institutional households [11]. However, the weakening of home care may lead to a common Chinese saying: “one disabled, a whole family unbalanced”. Bowen family system theory [12] states that the family is a system in which a change in one part of the system is followed by compensatory changes in other parts of the system. With abundant clinical observations and experiences, Bowen defined family functioning patterns as “overfunction and dysfunction” reciprocating mechanisms. For example, when a family member becomes temporarily ill, another family member will automatically overfunction to compensate for the dysfunction. However, when the illness develops into a chronic and fixed state and the family is unable to obtain the necessary help, overfunction may ultimately develop into dysfunction or even nonfunction, in which the family system loses flexibility will, leading to collapse.

A considerable number of studies have been published on the use of formal and informal care by community-dwelling older adults to assist with ageing in place [13]. There are mature models of formal care and the practice of formal care is mostly well known, such as the CAPABLE and PACE programs in the United States and the PRISMA program in Canada [14–17]. In 2018, the NHS in England changed the name of accountable care systems to integrated care systems, further emphasizing an integrated hospital-community-home care model for older adults with disabilities [18]. As informal care is mainly provided by family members, close relatives, friends or neighbours, there are no contracts for caregivers regarding care responsibilities and they are normally not paid [19]. Studies in the field of theoretical research seem to be more known. For example, based on the relationship between informal care and formal care, a series of theoretical models of care support have become famous, namely, the substitution model [20], task specificity model [21], complementary model [22], hierarchical compensatory model [23] and the covey of care model [24]. The Cantor hierarchical compensatory model points out that when primary informal care (for example, care provided by a child or spouse) and secondary informal care (for example, care provided by neighbours or friends) inadequate, tertiary quasi-formal (for example, from religious organizations) and peripheral formal support (for example, from voluntary and governmental service organizations) plays an essential role [23]. From the perspective of intergenerational relationships, academics have divided intergenerational relationships into different subtypes to identify the strength of the relationship, intergenerational support and emotional affinity between parents and child within a family [25]. For example, Huang used the theory of intergenerational solidarity to classify the relationships between adult children and parents in Chinese families into 3 classes, including (a) tight-knit, (b) supportive but distant, and (c) detached relationships [26]. Studies have also used clustering techniques to identify care network subtypes [27, 28] and observe the effects of different subtypes on care outcomes, such as nursing home placement [29].

To our knowledge, there is no research on the classification of hidden patterns of home care among families of older adults with disabilities in China. According to family system theory, we support that social support is necessary to maintain the dynamic balance of the family system and to avoid losing the flexibility and function of the family, thus ensuring its normal operation. Moreover, community support is a medium level of social support, which is valuable for integrating resources and providing specific services [30]. Nevertheless, China's home-based care service is still in the exploratory stage, and the policy is not perfect. For example, long-term care insurance started in 2016 and is still in the pilot stage in most cities [31]. On March 13, 2021, China launched its fourteenth National 5-Year Plan, which includes the objective to “support families in assuming the function of providing older adults care, and establish a system of older adults care services that coordinates care between the home and community institutions and simultaneously combines medical care with health care” [32]. This study used data from the Chinese Longitudinal Healthy Longevity Survey (CLHLS) with the latent class analysis (LCA) method to identify the types and characteristics of home care for older adults with disabilities in China, to explore family needs and preferences through subgroup analysis to improve the utilization and allocation efficiency of limited resources, in addition to helping shape public policy.

## Methods

### Data collection

This dataset is derived from the 8th wave of the CLHLS in 2018. The CLHLS aims to better understand the social, behavioural, environmental and biological factors that affect human longevity and health. It was supported by the United Nations Funds for Population Activities, the National Institute on Aging in America, and the National Natural Science Foundation of China [3, 7]. The first survey, carried out by Peking University in 1998, covered 22 of China's 31 provinces, including 85 percent of the country's population, in which Han Chinese people are the overwhelming majority [11]. Follow-up surveys were conducted every three to four years. Thus far, the CLHLS database is the largest longitudinal survey on the influencing factors of ageing on health in China and worldwide [33, 34]. Its quality has been unanimously supported by both domestic and foreign peer experts [35].

The survey uses a multistage nonproportional target random sampling method (a master sample of 10,000 oldest-old individuals aged 80 + years vs. a subsample of approximately 5,000 individuals aged 65–79 years). The practice is common in countless other countries, where similar surveys are conducted with older people who have lived long lives [34]. The survey thus oversampled extremely old persons and oversampled male oldest old individuals, given that there are fewer persons at more advanced ages, and fewer males than females [11]. Therefore, what makes this database specific is that it focuses more on the oldest-old persons aged 80 and above, which are most likely to need help.

The 2018 follow-up survey included 15,874 respondents aged 65 + years. In this study, the Katz scale [36] was used to extract data for 4638 older adults with disabilities, with the exclusion of 314 who resided in nursing homes. After deleting responses that were illogical or missing key variables, 3725 eligible responses were retained for final analysis. Among them, there were 1826 individuals with mild disability, accounting for 49.0% of the sample; 788 individuals with moderate disability, accounting for 21.2% of the sample; and 1111 individuals with severe disability, accounting for 29.8% of the sample. The data can be obtained free of charge from the Peking University Open Research Data platform.

### Measures

#### Manifest variables

LCA is a form of latent mixed variable modelling. In LCA, each latent variable is categorical and comprises a set of latent classes. Ruscio defined categorical latent variables as those in which "qualitative differences exist between groups of people or objects” [37]. These latent classes are measured by manifest indicators. The manifest variables are independent variables. We used family system theory, the literature and available relevant variables to guide our selection of manifest variables for LCA. Bowen believes that caregivers should receive the same attention in the family system as those who receive care [12]. A previous study reported that the degree of disability and the care duration are important factors influencing caregivers' performance [38, 39], and demand satisfaction can reflect the quality of care in a family [13]. Cantor points out that dynamic changes in family size affect the ability of families to provide care for older adults with disabilities [40]. Therefore, we comprehensively included five indicators as manifest variables, including the degree of disability, demand satisfaction, care duration, care performance, and the living status. It should also be noted that the inclusion of manifest variables is not achieved overnight. In addition to searching for characteristic indicators reported in the literature, we also conducted repeated model fitting multiple times to find the best combination scheme.

According to the six indicators of the Katz scale, including eating, dressing, defecation control, walking indoors, going to the toilet and bathing, the degree of disability was divided into three levels according to the number of items scored as "partially in need of help" or "completely in need of help"; an indication of 1–2 items rated as requiring help was defined as mild disability, an indication of 3–4 items requiring help was defined as moderate disability, and an indication of 5–6 items requiring help was classified as severe disability. According to whether the older adults with disabilities could meet their personal needs with help for the six Katz indicators, demand satisfaction was divided into three levels: "fully satisfied", "basically satisfied" and "dissatisfied". For care duration, the unit was days, and the longest duration of caregiver care for the above six indicators was taken as the length of providing care. Care performance was evaluated by the older adults who was receiving care. It was measured by asking the individual, "Do you think your primary caregiver shows the following performance in the care process"? The answer options included willing, impatient, incompetent, unwilling and unaware.

#### Outcome variables

We choose the preference for community support as our outcome variable under the guidance of Cantor's hierarchical compensatory theory. The CLHLS questionnaire measures community support using the following eight aspects: personal care, home visits, psychological consultations, daily shopping, social and recreational activities, legal aid, health education and neighbourhood relations. Older adults with disabilities were asked to answer two multiple-choice questions: (1) What kind of social services are available in your community? and (2) What kind of social services do you expect to be provided by your community? The answer was a binary variable, with responses of yes or no.

### Data analysis

Model selection in LCA can be challenging. Both overestimation and underestimation of the number of latent categories will affect the accuracy of the result inference. There are two critically important principles to consider: parsimony and model interpretability [41]. According to the principle of selecting the best fit model, a common approach is to use information criteria, such as Akaike’s information criterion (AIC), the Bayesian information criterion (BIC), the adjusted BIC (aBIC) and entropy. The Lo-Mendell-Rubin likelihood ratio (LMR LR) test and the bootstrap likelihood ratio test (BLRT) were used for significance testing in this study. In general, the smaller the AIC, BIC and aBIC values are, the better the model fits [42]. Entropy indicates classification accuracy. The value ranges from 0 to 1, and the closer the value is to 1, the more accurate the classification is [43]. An entropy level of 0.6 or higher provides sufficiently good class separation [44].

Once the optimal number of latent profiles was identified, older adults with disabilities were classified into latent classes. The latent classes were defined based on the conditional probability. Next, we were interested in exploring covariates to predict patterns of home care for older adults with disabilities in China. Are there differences in community support needs among families of older adults with disabilities with different care patterns? First, a latent multinomial logit model that regressed the latent class variable on major demographic characteristics and family factors (e.g., age, education level, marital status and rural/urban residence) was estimated using the R3STEP method [45]. Second, with the LCA model as the independent variable and community support needs as the outcome variable, Lanza’s method [46] was used to establish a mixed regression model. Third, the chi-square goodness-of-fit test was used to explore the care provisions for and preferences of the older adults with disabilities. All the models were constructed using Mplus 8.3. SPSS 25.0 was used to perform descriptive and chi-square goodness-of-fit tests.

## Results

### Descriptive analysis

Table 1 reports the descriptive statistics of the characteristics of the study sample. The mean age of the older adults with disabilities was 95.2 years (SD 8.519, range = 65–116 years). Of the 3725 participants, 49% had mild disability, 21.2% had moderate disability, and 29.8% had severe disability. A total of 32.6% of the participants were men. A total of 16.9% of the participants still had a spouse. The mean years of education was 1.9 years (SD 3.428, range = 0–20 years). The average objective physical performance test (3-item mini-PPT test) [11] score was 1.2 (SD 1.035, range = 0–3). There were 1982 participants (53.2%) living in the eastern region, 766 (20.5%) in the central region and 977 (26.2%) in the western region, including 2156 (57.9%) residing in cities and towns and 1569 (42.1%) residing in rural areas. The mean number of cohabitating persons living with older adults with disabilities was 2.17 persons per household (SD 1.746, range = 0–20 persons).

**Table 1:** Demographic characteristics and family characteristics (*n* = 3725)

Characteristics	Range/Catergory	Mean (SD)	N(%)
Age (years)	Range (65–116)	95.15 (8.519)	
Education (years)	Range (0–20)	1.90 (3.428)	
Physical performance^a^	Range (0–3)	1.20 (1.035)	
Number of cohabitating persons^b^	Range (0–20)	2.17 (1.746)	
Age group	65–79		225 (6.1)
	80–89		581 (15.6)
	90–99		1238 (33.2)
	≥ 100		1681 (45.1)
Gender	Male		1216 (32.6)
	Female		2509 (67.4)
Marital status^c^	Has a spouse		631 (16.9)
	No spouse		3094 (83.1)
Degree of disability	Mild disability		1826 (49.0)
	Moderate disability		788 (21.2)
	Severe disability		1111 (29.8)
Cognitive Function^d^	Good		997 (26.7)
	Moderate		323 (8.6)
	Poor		2405 (64.5)
Occupation	Agricultural		2689 (72.1)
	Non-agricultural		1036 (27.8)
Region	Eastern region		1982 (53.2)
	Central region		766 (20.5)
	Western region		977 (26.2)
Residence	Urban		2156 (57.9)
	Rural		1569 (42.1)

*SD* Standard deviation; Missing data: For ranked data, hot deck imputation was used; for measurement data, imputation was used

^a^3-item mini-Physical Performance Test (3-item mini-PPT): developed by Zeng [11], contains three tests: stand-up from a chair, pick-up a book from the floor, and turning approximately 360°. The total scores of the three tests range from 0 to 3, with higher scores indicating better physical function

^b^Number of cohabitating persons: excluding the older adults with disabilities

^c^Has a spouse: the spouse is alive; No spouse: divorced, unmarried or widowed

^d^Chinese version of the Mini-Mental State Examination (CMMSE), which was culturally translated and adapted into the Chinese language based on the international standard MMSE questionnaire. The same cut offs of the standard MMSE were used for measuring cognitive function: a score of 24–30 was defined as “Good”, a score of 21–23 was defined as “Moderate”, and a score < 21 was defined as “Poor” [11]

### Latent class analysis

Starting from the initial model with 1 potential category, a total of 5 potential category models were fitted (Table 2). The AIC, BIC and aBIC parameters of the different models were compared. The AIC of the model with four latent classes was the smallest, which suggested that this model was preferable, while the BIC and aBIC suggested that the model with three latent classes provided an optimal balance between fit and parsimony. Studies have demonstrated that the aBIC is the information index with the highest classification accuracy based on at least 50 subjects in each category [47]. Class 3, the smallest classification in this study, also had 344 subjects, so this criterion was applicable. Additionally, the entropy value showed that the three-class model had the best classification accuracy (entropy = 0.737). The LMR LR test and BLRT showed a statistically significant difference. Therefore, we concluded that the 3-class model was optimal.

**Table 2:** Latent class model fit comparison (*n* = 3725)

Model	df	AIC	BIC	aBIC	Entropy	LMR	BLRT	Prob
1	11	30999.185	31067.636	31032.683	-	-	-	1
2	23	30,660.697	30,803.822	30,730.739	0.582	0	0	0.241/0.759
**3**	35	30579.423	**30797.222**	**30686.009**	**0.737**	**0.0085**	**0**	0.468/0.439/0.092
4	47	**30559.584**	30852.056	30702.713	0.524	0.022	0	0.328/0.021/0.449/0.202
5	59	30565.261	30932.407	30744.934	0.549	1	1	0.291/0.010/0.215/0.180/0.498

*df* degrees of freedom, *AIC* Akaike information criterion, *BIC* Bayesian information criterion, *aBIC* adjusted BIC, *LMR LR* Lo-Mendell-Rubin likelihood ratio, *BLRT* bootstrap likelihood ratio test

-: Not applicable

After determining the best model, according to the conditional probability of the latent class model (Table 3), we named the three latent classes. Because 99.1% of the participants in Class 1 had mild disability and 95.8% of the caregivers were “willing to care", we defined this class as those with mild disability and strong care. In Class 2, because older adults with moderate and severe disabilities accounted for 43.0 and 57.0% of the group, respectively, and because 96.8% of caregivers were willing to take care of them, we defined this class as those with severe disability and strong care. In Class 3, individuals with moderate and severe disabilities accounted for certain proportions, but the members of this group mainly had severe disability, accounting for 52.0% of the group. Different from the first two groups, 55.2% of the caregivers in Class 3 were "incompetent"; the probability of fully meeting the older adults with disabilities needs was 0, and 22.7% of their care needs could not be met. Thus, this class was labeled as having severe disability and incompetent care.

**Table 3:** Patterns of home care for older adults with disabilities in China measured by latent class analysis: Selected results of the 3-class LCA

Observed Variables	Conditional Probability by Class		
	Class 1 (*n* = 1745, 46.85%)	Class 2 (*n* = 1636, 43.92%)	Class 3 (*n* = 344, 9.24%)
**Care Duration**			
Half a year	0.278	0.258	0.294
Half a year ~ Five years	**0.569**	**0.528**	**0.506**
≥ 5 years	0.153	0.214	0.201
**Degree of Disability**			
Mild disability	**0.991**	0.000	0.279
Moderate disability	0.009	0.430	0.201
Severe disability	0.000	**0.570**	**0.520**
**Care Performance**			
Willing	**0.958**	**0.968**	0.093
Impatient/unwilling	0.006	0.001	0.180
Incompetent	0.011	0.011	**0.552**
Unaware	0.025	0.021	0.174
**Demand satisfaction**			
Fully Satisfied	**0.606**	0.469	0.000
Basically Satisfied	0.394	**0.531**	**0.773**
Dissatisfied	0.000	0.000	0.227
**Living status**			
Lives alone	0.124	0.042	0.105
Two people live together	0.275	0.303	0.270
At least three people live together	**0.601**	**0.655**	**0.625**

n: number of subjects by class

Item-response probabilities > .5 are presented in bold to facilitate interpretation

Further analysis was performed on the composition of primary caregivers. Table 4 shows that offspring care is the predominated care model for older adults with disabilities (average of 81%). 2.7% of Class 1 individuals live alone (2.7%, *P* < 0.05). Class 2 group received the largest proportion of nannies and social services (10%, *P* < 0.05). The Class 3 group showed a higher proportion of relatives, friends and neighbours who provided care (3.2%, *P* < 0.05). The difference was statistically significant compared to the other two groups.

**Table 4:** Analysis results of the primary caregivers in the three different types of families

Caregivers	Class 1	Class 2	Class 3	Total
Unattended	**47a**	1b	0b	48
	**2.70%**	0.10%	0.00%	1.30%
Spousal care	181a	127b	39a, b	347
	10.40%	7.80%	11.30%	9.30%
Offspring care	1401a	1332a	284a	**3017**
	80.30%	81.40%	82.60%	**81.00%**
Relatives, friends and neighbours	23a	13a	**11b**	47
	1.30%	0.80%	**3.20%**	1.30%
Nannies and social services	93a	**163b**	10a	266
	5.30%	**10.00%**	2.90%	7.10%
Total	1745	1636	344	3725
	100.00%	100.00%	100.0%	100.00%

a and b represent pairwise comparisons, and the difference is statistically significant

### LCA with covariates

To identify characteristics that predicted membership in the various latent classes, we incorporated covariates into the LCA. A baseline LCA including covariates is called regression mixture modelling (RMM) [48]. We treated the latent class as an outcome variable and major demographic characteristics and family characteristics (e.g., age, education level, marital status and rural/urban residence) as independent variables. The results of RMM using the R3STEP method are shown in Table 5. Class 3 served as the reference category. The results show that compared to those of Class 3 (severe disability, incompetent care), the influencing factors of Class 1 (mild disability, strong care) were physical function, spouse, cognitive function, region and economic status.

**Table 5:** Analysis of influencing factors of family type division of older adults with disabilities in China

Covariates	Latent Class					
	Class 1(*n* = 1745)		Class 2(*n* = 1636)		Class 3(*n* = 344)	
	OR	95% CI	OR	95% CI	OR	95% CI
Physical performance	**4.173** ^**c**^	3.093–5.628	**0.459** ^**c**^	0.319–0.661	-	-
Marital status						
No spouse	-	-	-	-		
Has a spouse	**0.291** ^**c**^	0.154–0.553	0.628	0.338–1.166	-	-
Cognitive Function						
Good	-	-	-	-		
Moderate	0.495	0.178–1.374	0.704	0.241–2.051	-	-
Poor	**0.176** ^**c**^	0.086–0.362	0.578	0.278–1.201	-	-
Region						
Eastern region	-	-	-	-		
Central region	**0.517** ^**c**^	0.325–0.822	**0.502** ^**c**^	0.322–0.781	-	-
Western region	**0.458** ^**c**^	0.288–0.726	**0.429** ^**c**^	0.276–0.666	-	-
Self-perceived economic status^a^						
Insufficient	-	-	-	-		
Sufficient	**5.812** ^**c**^	3.767–8.967	**4.271** ^**c**^	2.880–6.334	-	-
Education level^b^	1.155	0.858–1.554	1.314	0.978–1.764	-	-
Occupation						
Agricultural	-	-	-	-		
Non-agricultural	1.108	0.621–1.967	1.702	0.979–2.959	-	-
Gender						
Male	-	-	-	-	-	-
Female	1.286	0.803–2.057	1.341	0.840–2.141		
Age group						
65–79	-	-	-	-		
80–89	1.115	0.400–3.111	1.473	0.470–4.621	-	-
90–99	1.423	0.521–3.883	1.606	0.533–4.838	-	-
≥ 100	0.913	0.327–2.552	1.273	0.415–3.906	-	-
Residence						
Rural	-	-	-	-	-	-
Urban	1.169	0.789–1.732	1.274	0.853–1.823	-	-

*OR* Odds ratio, *95% CI* 95% confidence interval of OR

-: Reference group

^a^Self-reported financial status was measured by asking the participants if they had enough money to meet their daily living expenses. The answer was sufficient or insufficient

^b^Education: used z-scale

^c^Statistically significant at the 0.05 level

Cognitive functions in Class 1 were better than those in Class 3, especially at the poor function level (OR = 0.176, 95% CI = 0.086–0.362). Class 3 had a higher number of surviving spouses. Geographically, members in the Class 1 group were mainly located in eastern China (central region OR = 0.517, 95% CI = 0.325–0.822; western region OR = 0.458, 95% CI = 0.288–0.726). Compared with Class 3, in Class 1, the physical performance score of older adults with disabilities was higher (OR = 4.173, 95% CI = 3.093–5.628). The individuals in the Class 1 group also had a higher economic status (OR = 5.812, 95% CI = 3.767–8.967).

Physical performance, region and economic status also influenced the individuals in the Class 2 group (severe disability, strong care). Compared with that of Class 3, the physical performance score of Class 2 was lower (OR = 0.459, 95% CI = 0.319- 0.661), and it was also the lowest among the three groups. However, similar to those in the Class 1 group, the members of the Class 2 group were mainly located in the wealthier eastern region of China (central region OR = 0.502, 95% CI = 0.322- 0.781; western region OR = 0.429, 95% CI = 0.276- 0.666). The economic status of the Class 2 group was also higher than that of the Class 3 group (OR = 4.271, 95% CI = 2.880- 6.334). However, we did not find education level, occupation, gender, age, or residence in rural areas to have any effect on the latent classes.

### LCA with category outcome variables

The relationships between latent class membership and the odds of receiving 8 types of community support were examined using Lanza’s method (Tables 6 and 7) [46]. As shown in Table 7, compared to the group with mild disability and strong care and the group with severe disability and strong care, the group with incompetent care (Class 3) showed a higher demand for "personal care". Specifically, the probability of the incompetent group “wishing for personal care from the community" was 0.760. The corresponding probabilities for the other groups were 0.639 and 0.671, respectively. There was a significant difference when comparing Class 3 with Class 1 and Class 2, while there was no difference between the Class 1 and Class 2 groups. There was no significant difference in other aspects of community support among the three groups.

**Table 6:** Overall comparison of community support requirements^a^ by latent class

Community Support	Chi-Square	*P* Value
**1. personal care**	**14.295** ^**b**^	**0.001**
2. home visit	1.294	0.524
3. psychological consultations	0.227	0.893
4. daily shopping	0.202	0.904
5. social and recreational activities	0.426	0.808
6. legal aid	1.035	0.596
7. health care education	2.526	0.283
8. neighbourhood relations	0.193	0.908

^a^Estimated using Lanza’s method [46]

^b^Statistically significant at the 0.05 level

**Table 7:** Estimated probability^a^ of personal care requirements by latent class

Classes	personal care Prob	S.E	Class 2		Class 3	
			Chi-Square	*P* Value	Chi-Square	*P* Value
Class 1	0.639	0.015	2.085	0.149	**11.817** ^**b**^	0.001
Class 2	0.671	0.015			**4.331** ^**b**^	0.037
Class 3	0.760	0.035				

^a^Estimated using Lanza’s method [46]

^b^Statistically significant at the 0.05 level

According to the 8 community care services items in the CLHLS questionnaire, multiple response analysis and the chi-square goodness-of-fit test were performed with SPSS 25.0: it was concluded that the families of older adults with disabilities prefer home visits from health professionals(91.7%, Res. = 527.3) and health care education(83.3%, Res. = 248.3), and the residual error was positive. Psychological consultations, daily shopping, social and recreational activities, legal aid, neighbourhood relations and personal care were not preferred, as the residual error was negative (chi square = 196.247, *P* < 0.05), and the difference was statistically significant. The community’s preferred form of support was health care education(65.1%, Res. = 700), home visits (56%, Res. = 484) and neighbourhood relations (46%, Res. = 245), and the residual error was positive. Psychological consultations, daily shopping, social and recreational activities, legal aid and personal care were not preferred, as the residual error was negative (chi square = 1555.972, *P* < 0.05), and the difference was statistically significant (Table 8).

**Table 8:** Overall supply and demand of community support for older adults with disabilities in China

Community support	Demand (%)	Residual-1	Supply (%)	Residual-2
Personal care	74.5	-43.8	16.3	-463
Home visit	91.7^a^	527.3	56.0^b^	484
Psychological consultations	75.1	-23.8	23.2	-299
Daily shopping	66.6	-307.8	16.2	-464
Social and recreational activities	71.4	-146.8	31.9	-92
Legal aid	70.6	-175.8	31.1	-111
Health care education	83.3^b^	248.3	65.1^a^	700
Neighbourhood relations	73.5	-77.8	46.0^c^	245
Chi-Square	196.247^d^		1555.972^d^	

Method: Multiple response analysis and chi-square goodness-of-fit test

Percentages and totals are based on responders

Supply and demand refer to the proportion of older adults who answered “yes”, regarding supply and demand in the total sample

^a, b, c^The residual was positive, and the percentages are sorted from high to low

^d^Statistically significant at the 0.05 level

## Discussion

This study explored different patterns of home care for older adults with disabilities and their preferences for community support. Three different types of home care patterns were identified based on the degree of disability, demand satisfaction, care duration, care performance and living status. The three identified latent classes were as follows: Class 1: mild disability and strong care (46.85%); Class 2: severe disability and strong care (43.92%); and Class 3: severe disability and incompetent care (9.24%). Previous studies have revealed that caregiver stress is predominated by disability levels; that is, the more serious the loss of an individual’s self-care ability is, the greater their demand for personal care [49], ultimately leading to additional negative performances [38]. In this study, individuals in Class 2 and Class 3 had more severe disabilities than those in Class 1. By logic, these two classes should be under the greatest pressure. In particular, Class 2 had the lowest physical performance score (OR = 0.459, 95% CI = 0.319–0.661). To our surprise, caregivers in Class 2 presented a brilliant strong care willingness and ability, similar to those with mild disability and strong care (Class 1). As mentioned earlier, an in-depth analysis of the composition of the caregivers among the three groups showed that, beyond the dominated offspring care model (average of 81%), Class 2 had the largest proportion of formal support from nannies and social services (10%, *P* < 0.05), while Class 3 showed a higher proportion of informal care from relatives, friends and neighbours (3.2%, *P* < 0.05). This phenomenon may support the "buffering effect" of social support in the relationship between care intensity and care performance proposed by previous scholars [38, 50]. In any case, this question merits further investigation to explore the mechanism of influence.

We discovered that financial factors and geographic distribution significantly affected the patterns among the three classes. Stabile states that two important elements in the care setting are those who pay and those who deliver care [51]. Chen also pointed out that the core issue for older adults receiving care is the source of pension funds, that is, who provides the pension funds. When the pension fund issue is solved, the urgency and difficulty of solving other problems will be greatly reduced [52]. Individuals in Classes 1 and 2 shared similar characteristics: these individuals were mainly located in the eastern region and had an obvious economic advantage compared to those in Class 3 (OR = 5.812, 95% CI = 3.767–8.967; OR = 4.271, 95% CI = 2.880–6.334, respectively). Individuals in Class 3 mainly lived in the central and western regions. There are obvious regional differences among the eastern, central and western regions in China [53]. In general, the economic level, living conditions and infrastructure in the central and western regions are relatively less developed than those in the eastern region. Various support facilities for older adults have been developed more effectively in the eastern region due to earlier and faster ageing. Thus, older adults in the eastern region are more likely to live with diseases and disabilities longer [35]. Feng et al. reported that living in Central and Western China is a disadvantage for ageing in place [54].

We also found that Class 1 included more individuals with normal cognition function, while Class 3 included individuals with poorer cognition function and a higher number of surviving spouses. Clipp evaluated caregivers for cognitively impaired older adults with chronic illnesses such as cancer and indicated that spouse caregivers caring for individuals with dementia have a higher burdensome emotional impact [55]. Older adults’ caregivers are more adversely affected by the caregiving role than younger adults’ caregivers [56] because they are more vulnerable and suffer from illnesses and poor health [57]. In Class 3, 22.7 percent of the individuals did not have their needs met at all. Some studies have reported that compared with receiving appropriate long-term care, having unmet care needs leads to a higher incidence of hospitalization, greater psychological stress, higher mortality and a greater probability of institutionalization [58, 59]. This study suggests that we should pay more attention to older adults included in Class 3 and those in Class 1 and Class 2 whose needs are basically met. Furthermore, Lanza’s method shows that individuals in Class 3 preferred that the “community provides personal care” more than the other two groups. Therefore, additional community support, such as personal care services, adult day care services and respite care, should be prioritized within this group to meet the preferences and support needs for both the care recipient and their caregivers. At the same time, adequate and sustainable public financing and basic hardware construction in the central and western regions should be strengthened to improve the social security system for older adult populations.

Overall, the chi-square goodness-of-fit test showed that older adults in the community preferred home visits (home-based medical professional services) and health education. A number of studies have shown the effects of home visits for older adults: maintaining independence at home helps to reduce the mortality rate by 17%, increases the possibility of living in the community by 23% [60–62], delays nursing home admission and is associated with better health outcomes [29]. Plöthner M et al. defined professional health education needs as a subcategory of informational needs, which is one of the major topics of informal caregivers’ preferences when caring for frail older adults [6]. From the perspective of supply trends, there is a certain degree of matching between demand and supply. However, the contradiction between supply and demand is still quite prominent from the point of view of total volume (Table 8).

In recent years, China has launched a large number of national policy initiatives and regulations focusing on home care. The Notice on Strengthening Home Medical Services for older adults, published in 2020 by the National Hospital Authority, encourages secondary and primary medical institutions to provide home medical services for older adults with disabilities, transitional patients who have been recently discharged from acute hospitals and end-stage patients [60]. However, there may be a dangerous bias in China. As noted in the 2018 World Bank Group report, a large number of empty beds exist, but new residential care facilities are continually being built, leading to a waste of resources [6]. Moreover, some community care centres have set up dining tables, daily care services, senior universities and so on, but most of these services are for independent older adults and are rarely for disabled individuals [61]. Thus, rebalancing the long-term care system and reallocating resources are urgent. We firmly suggest that limited public resources should be based on accurate assessment of older people’s preferences and be targeted towards those who are most in need of care and whose families are incompetent. According to the WHO integrated care concept and international experience [14–17, 63, 64], integrating medical and social services care, empowering families, strengthening the training of general practitioners and long-term care nurses, accelerating the promotion of long-term care insurance and developing the internet support system are among the top priorities for improving the well-being of older Chinese people.

### Limitations

This study had several limitations. First, this paper did not exclude the community care services obtained at the time of this study when incorporating the family care ability for older adults with disabilities. Consequently, it reflects the home care ability and needs under the existing community care situation. Although there is no purely objective response regarding the family’s care ability, we can still see the care status and the benefits of existing social support services for those families. Second, the data from the CLHLS 2018 are second-hand data, and the measurement of some variables based on the CLHLS data is vague. For example, home visiting comprises many dimensions; in addition to providing home visits and health care education, it also includes nutritional support, rehabilitation training, medication guidance, symptom care, wound care, and pipeline care [65]. Further research could concentrate on more details regarding health care.

## Conclusions

To the best of our knowledge, this is the first study analysing the hidden patterns of home care among families of older adults with disabilities in China. In addition, their community support preferences were evaluated. Through this study, we hope we can provide some theoretical or methodological inspiration for similar research on the development of informal and formal care in the future, especially for developing countries. These findings have important implications for government policies: (a) We should still focus on the medical and health care needs of disabled families, vigorously enhancing the capacity of primary medical care and health services and specifically strengthening home visit services. (b) We should focus on helping the families of older adults with disabilities in the central and western regions and formulate corresponding policies and support measures. (c) Community support should take the family as a unit, comprehensively assess frail older adults' inability to care for themselves and the capacity of caregivers to deliver the best care and meet the needs of the family according to the principle of matching supply and demand. It should be emphasized that this is only a part of our research. In the future, under the guidance of family theories, we will utilize discrete selection experiments and other methods to collect more detailed data and describe local area experiences in reference to the national media.

## Data Availability

This study was based on datasets from the 8th Wave of the Chinese Longitudinal Healthy Longevity Survey (CLHLS) 2018 from the Peking University Open Research Data platform (https://doi.org/10.18170/DVN/WBO7LK). Researchers can obtain these data after submitting a data use agreement to the CLHLS team.

## Abbreviations

CAPABL: Community Ageing in Place: Advancing Better Living for Elders
PACE: Program for All-inclusive Care for the older adults
PRISMA: Programme of Research to Integrate Services for the Maintenance of Autonomy
CLHLS: Chinese Longitudinal Healthy Longevity Survey
LCA: Latent class analysis
RMM: Regression mixture modelling
AIC: Akaike Information Criterion
BIC: Bayesian Information Criterion
aBIC: Adjusted BIC
LMR LR: Lo-Mendell-Rubin likelihood ratio
BLRT: Bootstrap likelihood ratio test
